# Supplementary material for: When combinations of humans and AI are useful: A systematic review and meta-analysis
Source: Nat Hum Behav. 2024 Oct 28;8(12):2293–303. doi: 10.1038/s41562-024-02024-1 (PMC11659167; doi:10.1038/s41562-024-02024-1)
Supplement: Supplementary file 1 — Supplementary Figs. 1–8, Tables 1–5, Methods and Results. [file 41562_2024_2024_MOESM1_ESM.pdf]

nature human behaviour

Supplementary information https://doi.org/10.1038/s41562-024-02024-1

When combinations of humans and AI are useful: A systematic review and meta-analysis

In the format provided by the authors and unedited

Supplementary Information

Contents

S1 Supplementary Methods **2**

S1.1 Types of Outcomes in Human-AI Systems 2

S1.2 Systematic Literature Review 3

S1.3 Data Collection and Coding 4

S1.4 Calculation of Effect Size 5

S2 Supplementary Results **8**

S2.1 Descriptive Statistics 8

S2.2 Effect Sizes for Additional Outcomes 10

S2.3 Heterogeneity Analyses 12

S2.4 Moderators for Additional Outcomes 13

S2.5 Scatterplots of Effect Sizes for Accuracy-Based Tasks 14

S2.6 Division of Labor 17

S2.7 Effect Sizes over Time 18

S2.8 Bias Tests 20

S1 Supplementary Methods

S1.1 Types of Outcomes in Human-AI Systems

Let H, AI, and HAI represent the performance of the human alone, AI alone, and human-AI combination, respectively.

Definition 1 (Human-AI Synergy). *The human-AI group outperforms both the human and the AI alone.*

$$HAI > \max(H, AI)$$

Definition 2 (Human Augmentation). *The human-AI group outperforms the human alone.*

$$HAI > H$$

Definition 3 (AI Augmentation). *The human-AI group outperforms the AI alone.*

$$HAI > AI$$

Definition 4 (Negative Synergy). *The human-AI group outperforms neither the human or AI alone.*

$$HAI < \min(H, AI)$$

S1.2 Systematic Literature Review

Database	Search String
ACM Digital Library (ACM DL)	[[Abstract: human] OR [Abstract: expert] OR [Abstract: participant] OR [Abstract: humans] OR [Abstract: experts] OR [Abstract: participants]] AND [[Abstract: ai] OR [Abstract: "artificial intelligence"] OR [Abstract: ml] OR [Abstract: "machine learning"] OR [Abstract: "deep learning"]] AND [[Abstract: collaborate] OR [Abstract: assist] OR [Abstract: aid] OR [Abstract: interact] OR [Abstract: help]] AND [[Abstract: "experiment"] OR [Abstract: "experiments"] OR [Abstract: "user study"] OR [Abstract: "user studies"] OR [Abstract: "crowdsourced study"] OR [Abstract: "crowdsourced studies"] OR [Abstract: "laboratory study"] OR [Abstract: "laboratory studies"]] AND [E-Publication Date: (01/01/2020 TO 06/30/2023)]
Web of Science Core Collection (WoS)	(((AB=(human OR expert OR participant OR humans OR experts OR participants)) AND AB=(AI OR "artificial intelligence" OR ML OR "machine learning" OR "deep learning")) AND AB=(collaborate OR assist OR aid OR interact OR help)) AND AB=("experiment" OR "experiments" OR "user study" OR "user studies" OR "crowdsourced study" OR "crowdsourced studies" OR "laboratory study" OR "laboratory studies") Index Date 2020-01-01 to 2023-06-30
Association for Information Systems eLibrary (AISeL)	abstract:(human OR expert OR participant OR humans OR experts OR participants) AND abstract:(AI OR "artificial intelligence" OR ML OR "machine learning" OR "deep learning") AND abstract:(collaborate OR assist OR aid OR interact OR help) AND abstract:("experiment" OR "experiments" OR "user study" OR "user studies" OR "crowdsourced study" OR "crowdsourced studies" OR "laboratory study" OR "laboratory studies") Date Range = (01/01/2020 TO 06/30/2023)

Table S1: Syntax of the search strings for the literature review.

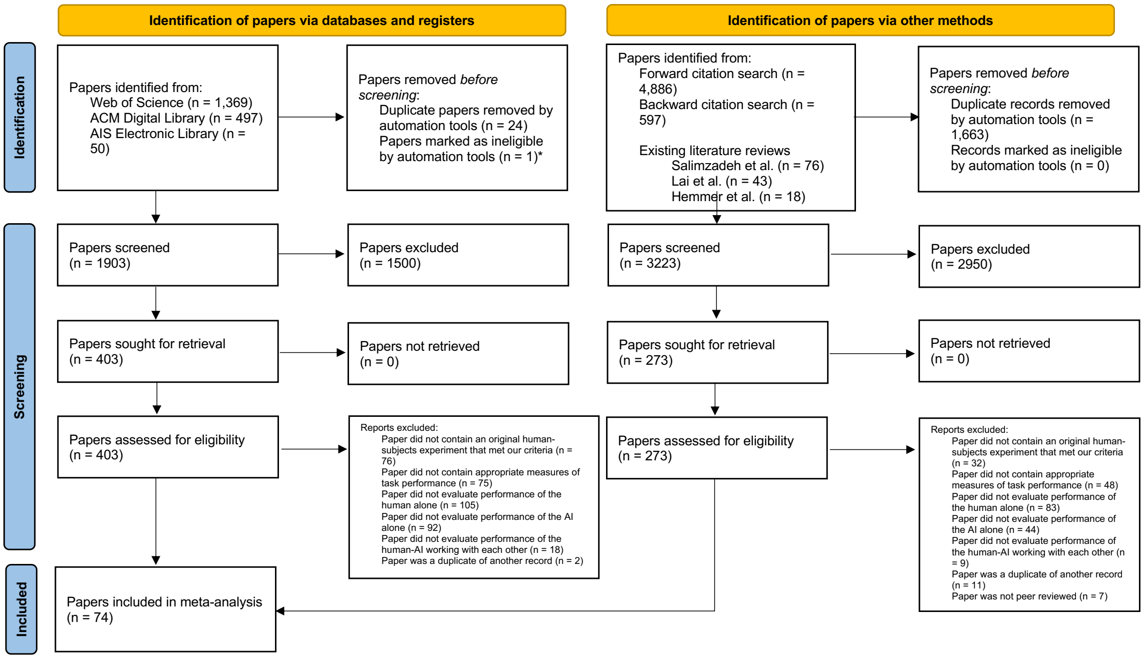

Figure S1: PRISMA flow diagram for the literature review and study inclusion process. *Article retracted from journal. Adapted from [1].

S1.3 Data Collection and Coding

We conducted the search in each of these databases in July 2023. We downloaded all search results as .ris files and imported them into Zotero to check for duplicates. Then, we exported the de-duplicated records into an Excel spreadsheet for screening. We extracted the records that passed our initial abstract screen into a separate sheet in the Excel file. Next, we evaluated if these records met our inclusion criteria by closely reading the paper. If they did, we put them into a separate Excel file for data extraction.

To calculate our primary outcome of interest – the effect of combining human and artificial intelligence on task performance – we recorded the averages and standard deviations of the task performance of the human alone, the AI alone, and the human and AI working with each other, as well as the number of subjects in each of these conditions. For example, in an experiment that involves N participants completing M trials (i.e., classifying M images) and that evaluates the task performance according to some metric x, we extracted the following values:

$$\mu_P = \frac{1}{N}\sum_{i=1}^{N} x_{P,i}$$

$$\sigma_P = \sqrt{\frac{\sum_{i=1}^{N}(x_{P,i} - \bar{x_P}}{N}}$$

Where P refers to the task performance of the human alone, the AI alone, or the human and AI working with each other:

$$P \in \{H, AI, HAI\}$$

And $x_{P,i}$ represents the task performance for participant i, defined as the average performance across the M trials:

$$x_{P,i} = \frac{1}{M}\sum_{i=1}^{M} x_{P,i,j}$$

As these formulas make clear, we selected individual humans for our primary unit of analysis as we designed our study to capture patterns in human behavior, particularly how they work with AI tools.

Many authors reported all of these values directly in the text of the paper. A notable number, however, reported them indirectly by providing 95% confidence intervals or standard errors instead of the raw standard deviations. For these, we calculated the standard deviations using the appropriate formulas [2].

Additionally, multiple papers did not provide the exact numbers needed for such formulas, but the authors made the raw data of their study publicly accessible. In these cases, we download the datasets and computed the averages and standard deviations using Python or R. If relevant data were only presented in the plots of a paper, we contacted the corresponding author to ask for the numeric values. If the authors did not respond, we used a Web Plot Digitizer [3] to convert plotted values into numerical values.

For papers that conducted an experiment that met our inclusion criteria but did not report all values need to calculate the effect size, we contacted the corresponding author directly to ask for the necessary information. If the author did not respond, we could not compute the effect size for the study and did not include it in our analysis.

We also considered and coded for multiple potential moderators of human-AI performance, as described in Table S2.

Many papers conducted multiple experiments, contained multiple treatments, or evaluated performance according to multiple measures. In such cases, we assigned a unique experiment identification number, treatment identification number, and measure identification number to the effect sizes from the paper. Note that we defined experiments based on samples of different sets of participants.

S1.4 Calculation of Effect Size

For each evaluation metric, we noted whether higher or lower values indicate better or worse performance on the task. For example, increases in the accuracy of a task indicate performances improvements, while increases the time to complete a task indicate performance losses. In the latter cases, we multiplied the performances of the human, AI, and human-AI combination by negative one for consistent interpretation of effect sizes consistent across our dataset.

As such, when Hedges' g equals zero, the average performance of the human-AI system and the baseline are the same, which indicates no gains from human-AI collaboration. A positive value of Hedges' g means that the average performance of the human-AI system exceeds that of the baseline, which points to synergy between humans and AI. In contrast, a negative value of Hedges' g means that the average performance of the human-AI system falls below that of the baseline, which points to performance losses from human-AI collaboration. Larger absolute values of Hedges' g indicate larger effects of human-AI collaboration on task performance. According to conventional interpretations, values of Hedges' g around 0.2 correspond to a small effect, values around 0.5 a medium effect, and values around 0.8 a large effect [4].

Moderator	Description
*Publication Date	Year the paper was published (2020, 2021, 2022, 2023)
*Experimental Design	Type of design of the experiment<br>1. Between-subjects<br>2. Within-subjects<br>3. Mixed, between-subjects (factorial design where the human vs. human-AI and AI vs. human-AI comparisons were the between-subjects factor)<br>4. Mixed, within-subjects (factorial design where the human vs. human-AI and AI vs. human-AI comparisons were the within-subjects factor)<br>*Note that in our moderator analysis, we combined "Between-subjects" and "Mixed, between-subjects" (and likewise "Within-subjects" and "Mixed, within-subjects").*
*Data Type	Type of data involved in the experimental task (Binary, Categorical, Image, Numeric, Text, Video)<br>*Note the if there were multiple types of data involved in the experiment (i.e. an image and a piece of text), we recorded them both (i.e. "Image, Text").*
*Task Type	Type of task evaluated<br>1. Creation: Does it involve some type of open response?<br>2. Decision: Does it involve deciding between a set of options or providing a numerical value?
*AI Type	Type of AI involved in the experiment<br>1. Shallow model<br>2. Deep learning model<br>3. Wizard of Oz model<br>*Note that, here, we follow Lai et al.'s classification scheme [5].*
*AI Explanation	Whether or not an explanation of the AI model's output was communicated to participants (Yes, No)
*Participant Type	Type of participant involved in the experiment<br>1. Crowdworker / non-crowdworker<br>2. Expert / non-expert
*Division of Labor	Was there some pre-determined division of labor between the human and AI?
Task Output	Type of output from the task (Binary, Categorical, Image, Numeric, Text, Video)
AI Confidence	Whether or not the confidence of the AI model was communicated to participants (Yes, No)
Who Performed Better?	Who performed better alone on the task (Human, AI)

Table S2: Descriptions of the moderator variables. *Indicates a pre-registered moderator variable.

S2 Supplementary Results

S2.1 Descriptive Statistics

Figure S2 provides the key descriptive statistics of the effect sizes in our data set. To highlight a few key takeaways, the vast majority come from experiments that evaluated human-AI performance in the context of a decision task, which often involves participants choosing between a set of options or providing a numerical value. Far fewer come from creation tasks, which entail some kind of open-ended response ($n = 34$, 9%). An even smaller number of effect sizes ($n = 4$, 1%) involve a pre-defined division of labor between the humans and AI tools, despite the potential for this process to lead to performance improvements.

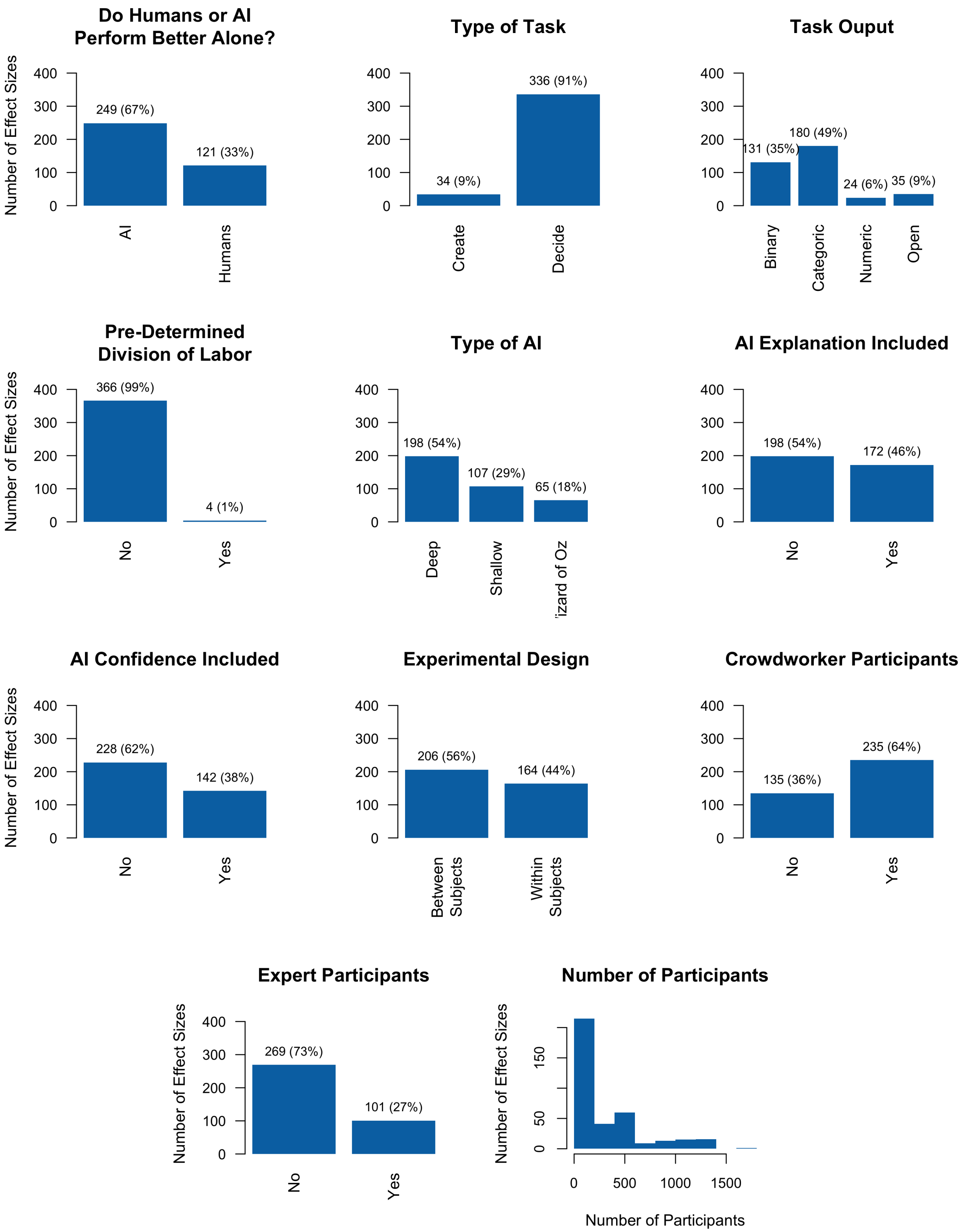

Figure S2: Descriptive statistics for the effect sizes in our analysis.

S2.2 Effect Sizes for Additional Outcomes

Measure	Human-AI Synergy	Human Augmentation	AI Augmentation	Negative Synergy
n	370	370	370	370
Hedges' g	-0.23	0.64	0.30	1.14
95% CI	[-0.39, -0.07]	[0.53, 0.74]	[-0.03, 0.62]	[0.90, 1.38]
t-statistic	$t(92.39) = -2.89$	$t(98.31) = 11.88$	$t(99.19) = 1.82$	$t(101.12) = 9.58$
p	0.005	0.000	0.072	0.000

Table S3: We fit separate meta-analytic models on the full set of results, and we report the results for human-AI synergy, human augmentation, AI augmentation, and negative synergy in this table. Here, n corresponds to the number of effect sizes in the model, Hedges' g is the meta-analytic average effect size, 95% CI refers to the 95% confidence interval for the effect size, t-statistic is the test statistic with the degrees of freedom estimated according to the Satterthwaite approximation, and p is the two-sided p-value that indicates if the effect size is statistically significant from zero.

	Human-AI Synergy		Human Augmentation	
Better Alone	AI	Human	AI	Human
n	249	121	249	121
Hedges' g	-0.53	0.39	0.73	0.39
95% CI	[-0.71, -0.35]	[0.21, 0.58]	[0.62, 0.84]	[0.20, 0.58]
t-statistic	$t(69.73) = -5.79$	$t(36.33) = 4.27$	$t(79.38) = 12.81$	$t(36.39) = 4.23$
p	0.000	0.000	0.000	0.000

Table S4: We fit separate meta-analytic models on the subset of results where (1) the AI performs better alone and (2) the human performs better alone, and we report the results for human-AI synergy and human augmentation in this table. Here, n corresponds to the number of effect sizes in the model, Hedges' g is the meta-analytic average effect size, 95% CI refers to the 95% confidence interval for the effect size, t-statistic is the test statistic with the degrees of freedom estimated according to the Satterthwaite approximation, and p is the two-sided p-value that indicates if the effect size is statistically significant from zero.

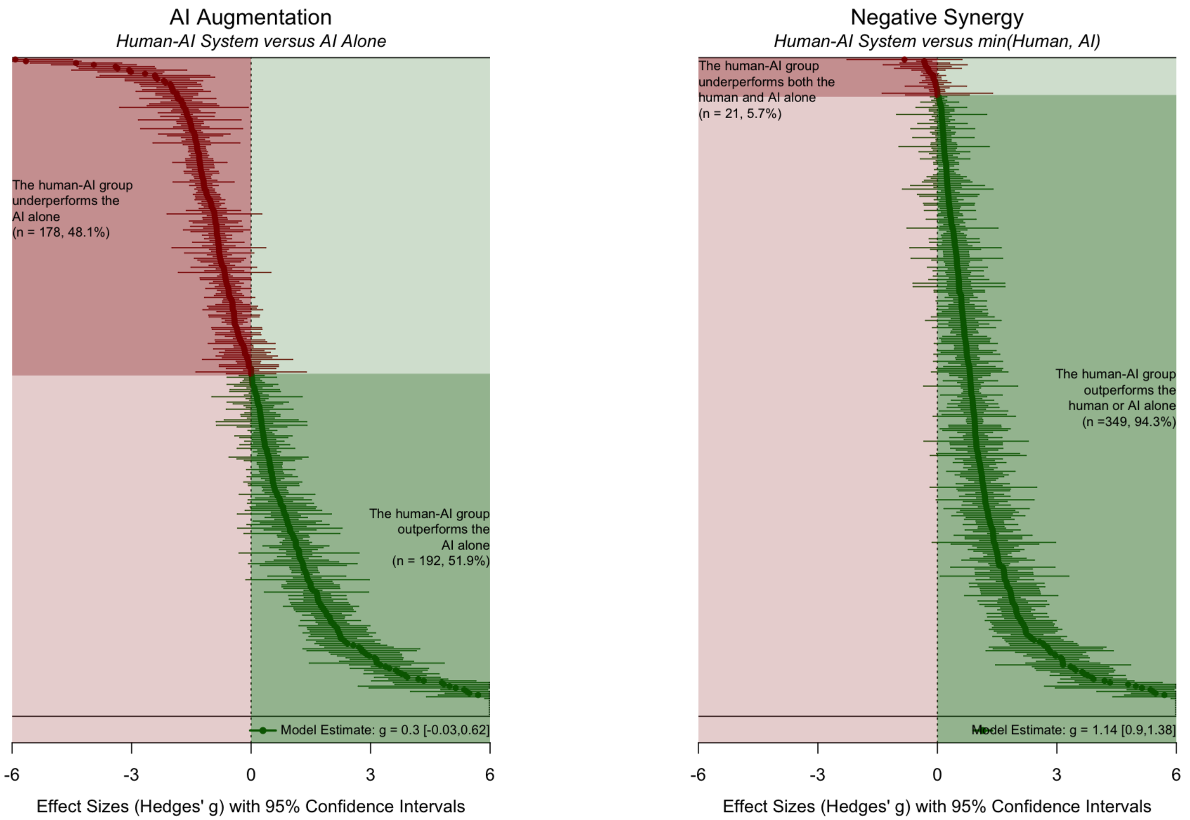

Figure S3: Forest plots of all effect sizes ($n = 370$) included in the meta-analysis for AI augmentation and negative synergy. The positions of the points on the x-axes represent the values of the effect sizes, and the bars indicate the 95% confidence interval for the effect sizes. The colors of the points and lines correspond to the values of the effect sizes, with negative effect sizes colored red and positive effect sizes colored green. For AI augmentation (a), a negative effect size means that the human-AI group performed worse than the AI alone, so there was no AI augmentation, and a positive effect size means that the human-AI group performed better than the AI alone, so there was AI augmentation. For negative synergy (b), a negative effect size means that the human-AI group performed worse than the AI alone and the human alone, so there was negative synergy, and a positive effect size means that the human-AI group performed better than the AI alone or the human alone, so there was not negative synergy. The black dotted line corresponds to an effect size of Hedges' $g = 0$, which means that the human-AI group performed the same as the baseline. The circle at the bottom of the graph represents the meta-analytic average effect size and confidence interval.

S2.3 Heterogeneity Analyses

Measure	Human-AI Synergy	Human Augmentation	AI Augmentation	Negative Synergy
n	370	370	370	370
τ^2	1.19	0.40	4.25	2.02
I^2	97.7%	93.8%	99.3%	98.6%
$I^2_{(2)}$	23.9%	43.6%	40.7%	50.1%
$I^2_{(3)}$	73.8%	50.2%	58.6%	48.5%
Q-Statistic	$Q(369) = 8532$	$Q(369) = 4226$	$Q(369) = 16017$	$Q(369) = 7209$
p	0.000	0.000	0.000	0.000

Table S5: We fit separate meta-analytic models on the full set of results, and we report the heterogeneity metrics for the models of human-AI synergy, human augmentation, AI augmentation, and negative synergy. In particular, we provide n (number of effect sizes), τ^2, I^2, $I^2_{(2)}$, $I^2_{(3)}$, Q-statistic (with the corresponding degrees of freedom), and p is the two-sided p-value from the Q-Test.

	**Human-AI Synergy**		**Human Augmentation**	
Better Alone	AI	Human	AI	Human
n	249	121	249	121
τ^2	1.25	0.49	0.31	0.49
I^2	98.1%	92.7%	93.0%	93.2%
$I^2_{(2)}$	17.9%	30.2%	52.5%	30.7%
$I^2_{(3)}$	80.2%	62.5%	40.5%	62.6%
Q-statistic	$Q(248) = 6111$	$Q(120) = 1071$	$Q(248) = 2741$	$Q(120) = 1136$
p	0.000	0.000	0.000	0.000

Table S6: We fit separate meta-analytic models on the subset of results where (1) the AI performs better alone and (2) the human performs better alone, and we report the heterogeneity metrics for the models of human-AI synergy and human augmentation. In particular, we provide n (number of effect sizes), τ^2, I^2, $I^2_{(2)}$, $I^2_{(3)}$, Q-statistic (with the corresponding degrees of freedom), and p is the two-sided p-value from the Q-Test.

S2.4 Moderators for Additional Outcomes

		Human-AI Synergy		Human Augmentation		AI Augmentation		Negative Synergy	
Subgroup	*n*	*Hedge's g*	*p*	*Hedge's g*	*p*	*Hedge's g*	*p*	*Hedge's g*	*p*
*^All Effect Sizes	370	-0.23 [-0.39, -0.07]	0.005	0.63 [0.53, 0.74]	0.000	0.30 [-0.03, 0.62]	0.072	1.14 [0.90, 1.38]	0.000
*^Who Performs Better Alone?									
AI	249	-0.54 [-0.71, -0.37]	0.000	0.74 [0.63, 0.85]	0.000	-0.53 [-0.74, -0.32]	0.000	0.76 [0.59, 0.92]	0.000
Human	121	0.46 [0.28, 0.65]	0.000	0.40 [0.25, 0.54]	0.000	2.20 [1.64, 2.76]	0.000	2.05 [1.54, 2.55]	0.000
*Type of Task									
Create	34	0.19 [-0.09, 0.48]	0.180	0.52 [0.11, 0.93]	0.013	1.07 [0.65, 1.49]	0.000	1.19 [0.87, 1.51]	0.000
Decide	336	-0.27 [-0.44, -0.10]	0.002	0.65 [0.54, 0.76]	0.000	0.22 [-0.14, 0.57]	0.223	1.13 [0.88, 1.39]	0.000
*Task Output									
Binary	132	-0.53 [-0.76, -0.31]	0.000	0.56 [0.40, 0.72]	0.000	-0.17 [-0.47, 0.13]	0.252	1.10 [0.78, 1.43]	0.000
Categoric	180	0.11 [-0.17, 0.39]	0.441	0.70 [0.52, 0.88]	0.000	0.87 [0.40, 1.34]	0.000	1.22 [0.72, 1.71]	0.000
Numeric	24	-0.81 [-1.20, -0.43]	0.000	0.91 [0.62, 1.21]	0.000	-0.77 [-1.19, -0.34]	0.000	0.91 [0.60, 1.22]	0.000
Open Response	34	0.19 [-0.09, 0.48]	0.186	0.52 [0.11, 0.93]	0.014	1.06 [0.64, 1.49]	0.000	1.19 [0.87, 1.51]	0.000
*^Task Data									
Image	164	0.07 [-0.22, 0.36]	0.647	0.84 [0.64, 1.03]	0.000	0.69 [0.23, 1.16]	0.004	1.26 [0.81, 1.71]	0.000
Multiple	78	-0.55 [-0.85, -0.25]	0.000	0.52 [0.27, 0.77]	0.000	-0.34 [-0.66, -0.02]	0.036	1.11 [0.60, 1.61]	0.000
Numeric	24	-1.08 [-1.28, -0.89]	0.000	0.84 [0.61, 1.06]	0.000	-1.05 [-1.26, -0.85]	0.000	0.83 [0.60, 1.05]	0.000
Text	96	-0.17 [-0.47, 0.13]	0.255	0.41 [0.22, 0.60]	0.000	0.63 [0.01, 1.26]	0.048	1.13 [0.69, 1.57]	0.000
Video	8	-0.03 [-0.39, 0.32]	0.847	0.25 [0.05, 0.45]	0.017	0.46 [-0.10, 1.02]	0.110	0.72 [0.38, 1.07]	0.000
*Year									
2020	68	-0.56 [-0.90, -0.21]	0.002	0.64 [0.30, 0.98]	0.000	-0.21 [-0.80, 0.39]	0.493	1.03 [0.65, 1.41]	0.000
2021	107	-0.47 [-0.76, -0.18]	0.002	0.60 [0.46, 0.74]	0.000	-0.11 [-0.51, 0.29]	0.576	1.14 [0.80, 1.47]	0.000
2022	130	0.01 [-0.39, 0.42]	0.954	0.58 [0.39, 0.77]	0.000	0.82 [0.30, 1.34]	0.002	1.08 [0.62, 1.54]	0.000
2023	65	0.11 [-0.21, 0.44]	0.488	0.81 [0.53, 1.09]	0.000	0.67 [-0.03, 1.36]	0.061	1.34 [0.83, 1.86]	0.000
*AI Type									
Deep	198	-0.02 [-0.27, 0.24]	0.897	0.60 [0.45, 0.75]	0.000	0.61 [0.30, 0.92]	0.000	1.04 [0.77, 1.32]	0.000
Shallow	107	-0.59 [-0.85, -0.33]	0.000	0.58 [0.42, 0.75]	0.000	-0.32 [-0.65, 0.01]	0.054	1.04 [0.69, 1.40]	0.000
Wizard of Oz	65	-0.15 [-0.64, 0.35]	0.559	0.82 [0.56, 1.09]	0.000	0.55 [-0.59, 1.68]	0.343	1.53 [0.69, 2.37]	0.000
^Experimental Design									
Dependent Samples	198	-0.17 [-0.40, 0.06]	0.041	0.74 [0.60, 0.88]	0.000	0.38 [-0.11, 0.87]	0.155	1.29 [0.93, 1.65]	0.000
Independent Samples	172	-0.29 [-0.50, -0.09]	0.009	0.52 [0.38, 0.67]	0.000	0.20 [-0.19, 0.60]	0.081	0.97 [0.70, 1.23]	0.000
AI Explanation Included									
No	228	-0.21 [-0.41, -0.01]	0.008	0.65 [0.52, 0.78]	0.000	0.31 [-0.12, 0.75]	0.247	1.17 [0.85, 1.49]	0.000
Yes	142	-0.25 [-0.43, -0.06]	0.212	0.62 [0.51, 0.73]	0.000	0.28 [-0.04, 0.59]	0.063	1.11 [0.90, 1.32]	0.000
AI Confidence Included									
No	269	-0.29 [-0.50, -0.08]	0.019	0.66 [0.52, 0.81]	0.000	0.26 [-0.18, 0.69]	0.092	1.19 [0.87, 1.50]	0.000
Yes	101	-0.12 [-0.31, 0.07]	0.098	0.59 [0.47, 0.71]	0.000	0.37 [-0.02, 0.76]	0.513	1.06 [0.77, 1.34]	0.000
Expert Participants									
No	135	-0.23 [-0.42, -0.04]	0.257	0.63 [0.49, 0.76]	0.000	0.37 [-0.06, 0.80]	0.166	1.18 [0.87, 1.50]	0.000
Yes	235	-0.23 [-0.51, 0.04]	0.006	0.67 [0.51, 0.83]	0.000	0.12 [-0.24, 0.48]	0.205	1.03 [0.81, 1.25]	0.000
Crowdworker Participants									
No	206	-0.14 [-0.38, 0.10]	0.146	0.66 [0.52, 0.80]	0.000	0.28 [-0.12, 0.67]	0.126	1.09 [0.82, 1.35]	0.000
Yes	164	-0.29 [-0.50, -0.08]	0.006	0.62 [0.47, 0.78]	0.000	0.31 [-0.17, 0.79]	0.311	1.18 [0.82, 1.54]	0.000
Divison of Labor									
No	366	-0.24 [-0.40, -0.08]	0.004	0.64 [0.54, 0.75]	0.000	0.29 [-0.05, 0.62]	0.090	1.14 [0.90, 1.38]	0.000
Yes	4	0.22 [-0.42, 0.87]	0.494	0.16 [-0.43, 0.74]	0.595	0.95 [0.69, 1.21]	0.000	0.91 [0.71, 1.12]	0.000

Table S7: Results from the three-level meta-regression models for the moderator variables. Here, n is the number of included effect sizes for the moderator subgroup level, g is the estimated effect size with the corresponding 95% confidence interval for the subgroup level, and p is the two-sided p-value of the estimated effect size for the subgroup level, which tests if the effect size is significantly different from zero.

S2.5 Scatterplots of Effect Sizes for Accuracy-Based Tasks

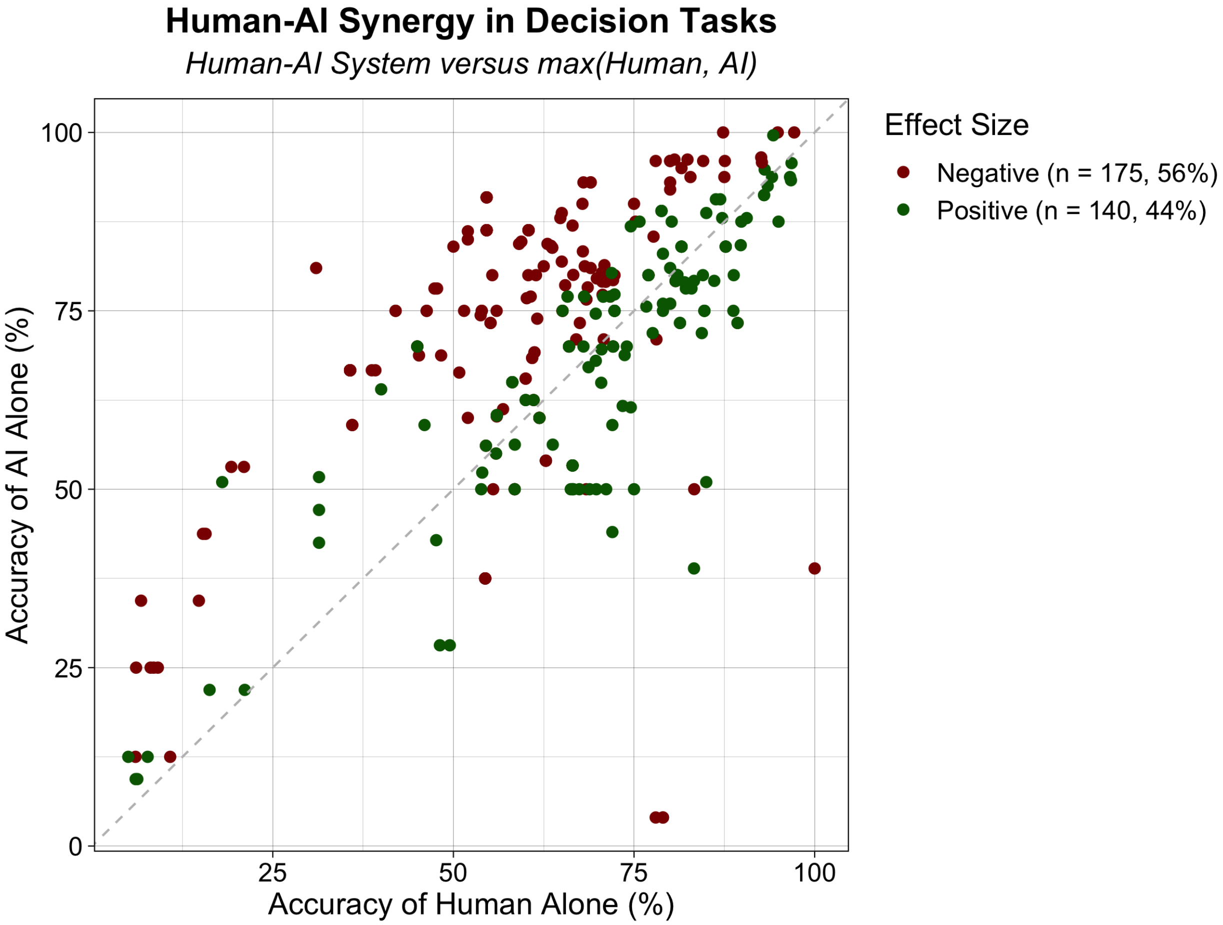

Figure S4: Scatterplot of the effect sizes that correspond to decision tasks where the researchers measured performance according to accuracy. The position of the points on the x-axis represents the accuracy of the human alone and the position of the points on the y-axis represents the accuracy of the AI alone. The colors of the points correspond to the value of the effect sizes, with negative effect sizes red and positive effect sizes green. A negative effect size means that the human-AI group performed worse than the human alone or the AI alone, so there was not human-AI synergy, and a positive effect size means that the human-AI group performed better than the human alone and the AI alone, so there was human-AI synergy. The gray dotted line indicates that the accuracy of the humans alone and AI alone are equivalent. In the region above this line, the accuracy of the AI alone exceeds that of the human alone, and we observe a large proportion of red points – no evidence of human-AI synergy. In the region below this line, the accuracy of the humans alone exceeds that of the AI alone, and we observe a large proportion of green points – evidence human-AI synergy.

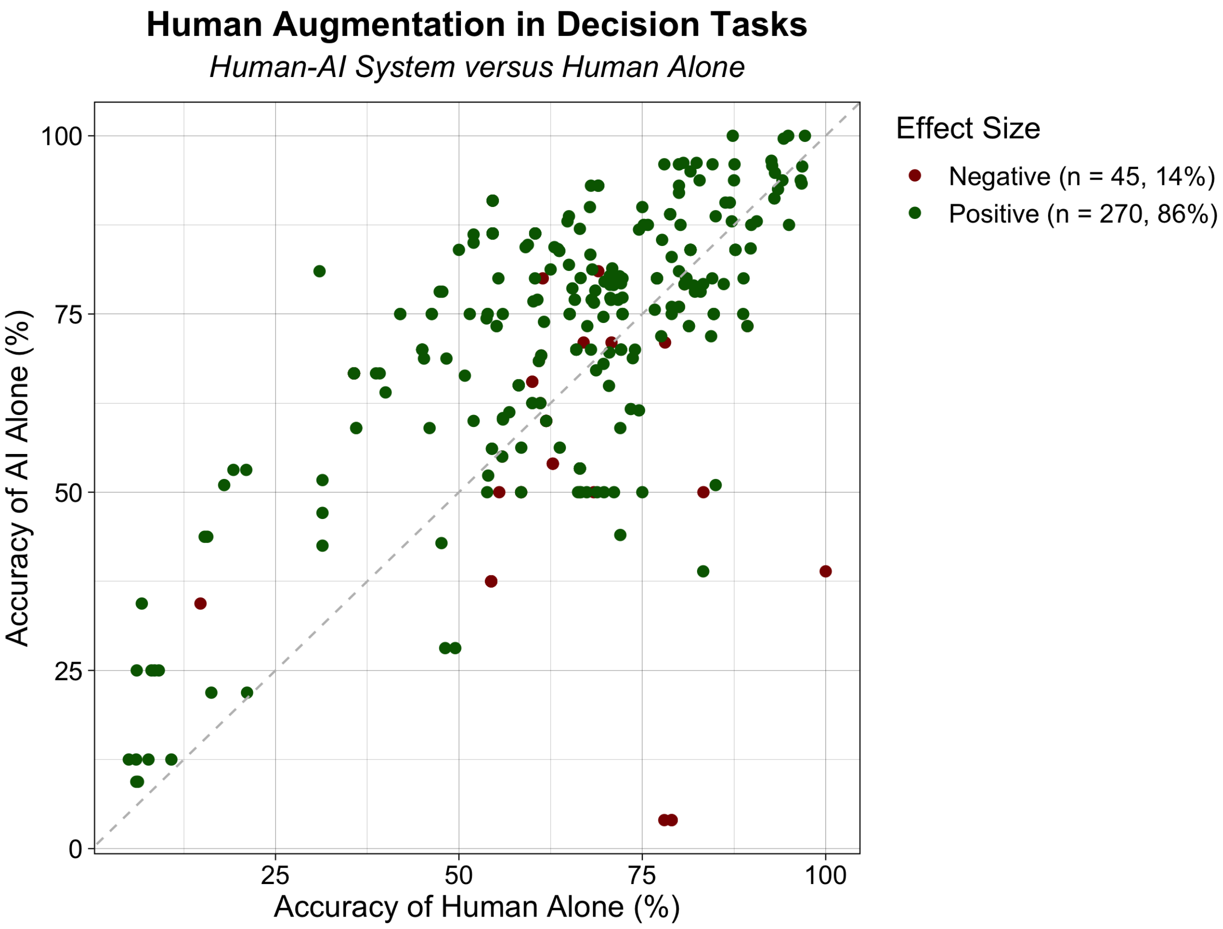

Figure S5: Scatterplot of the effect sizes that correspond to decision tasks where the researchers measured performance according to accuracy. The position of the points on the x-axis represents the accuracy of the human alone and the position of the points on the y-axis represents the accuracy of the AI alone. The colors of the points correspond to the value of the effect sizes, with negative effect sizes red and positive effect sizes green. A negative effect size means that the human-AI group performed worse than the human alone, so there was not human augmentation, and a positive effect size means that the human-AI group performed better than the human alone, so there was human augmentation. The gray dotted line indicates that the accuracy of the humans alone and AI alone are equivalent.

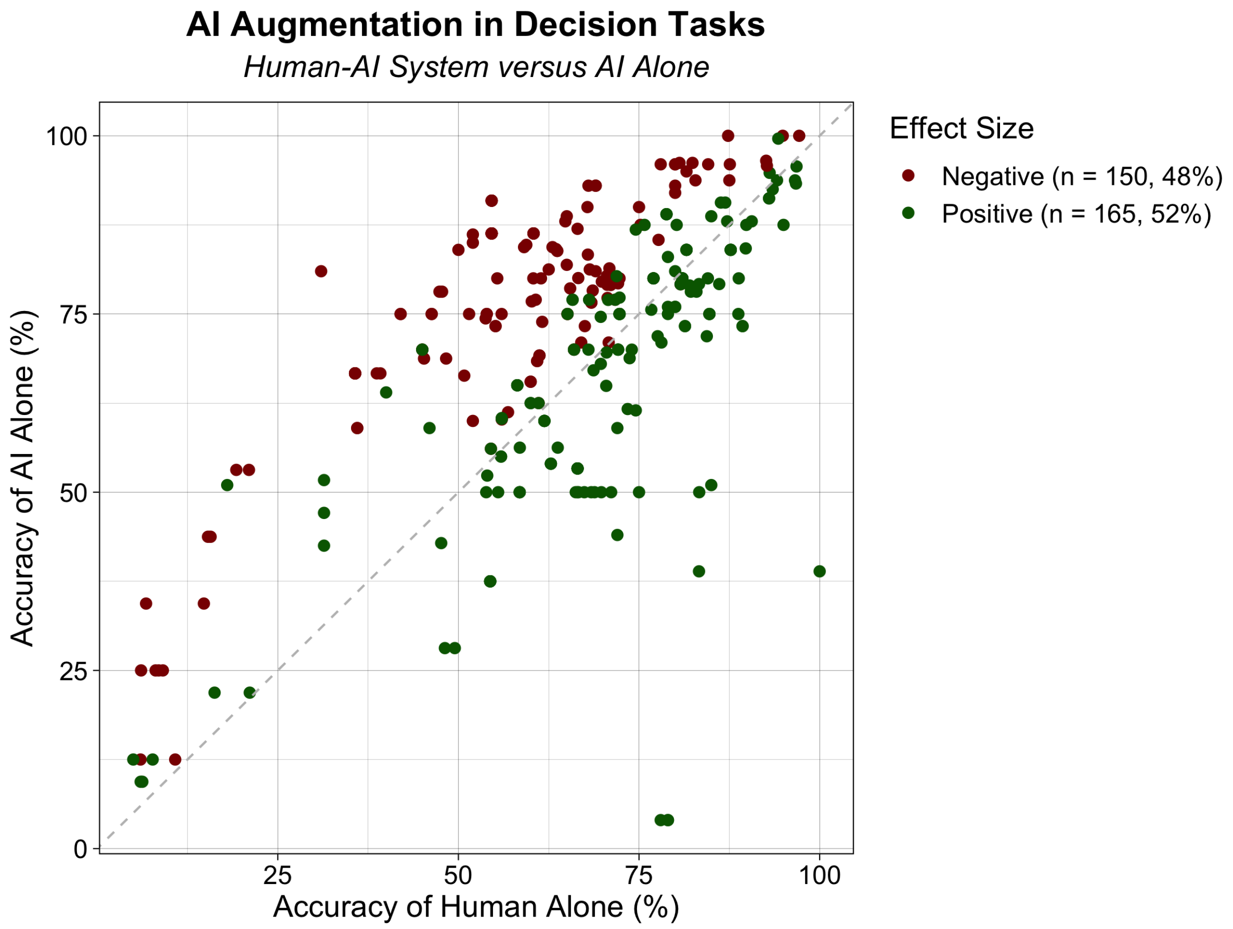

Figure S6: Scatterplot of the effect sizes that correspond to decision tasks where the researchers measured performance according to accuracy. The position of the points on the x-axis represents the accuracy of the human alone and the position of the points on the y-axis represents the accuracy of the AI alone. The colors of the points correspond to the value of the effect sizes, with negative effect sizes red and positive effect sizes green. A negative effect size means that the human-AI group performed worse than the AI alone, so there was not AI augmentation, and a positive effect size means that the human-AI group performed better than the AI alone, so there was AI augmentation. The gray dotted line indicates that the accuracy of the humans alone and AI alone are equivalent.

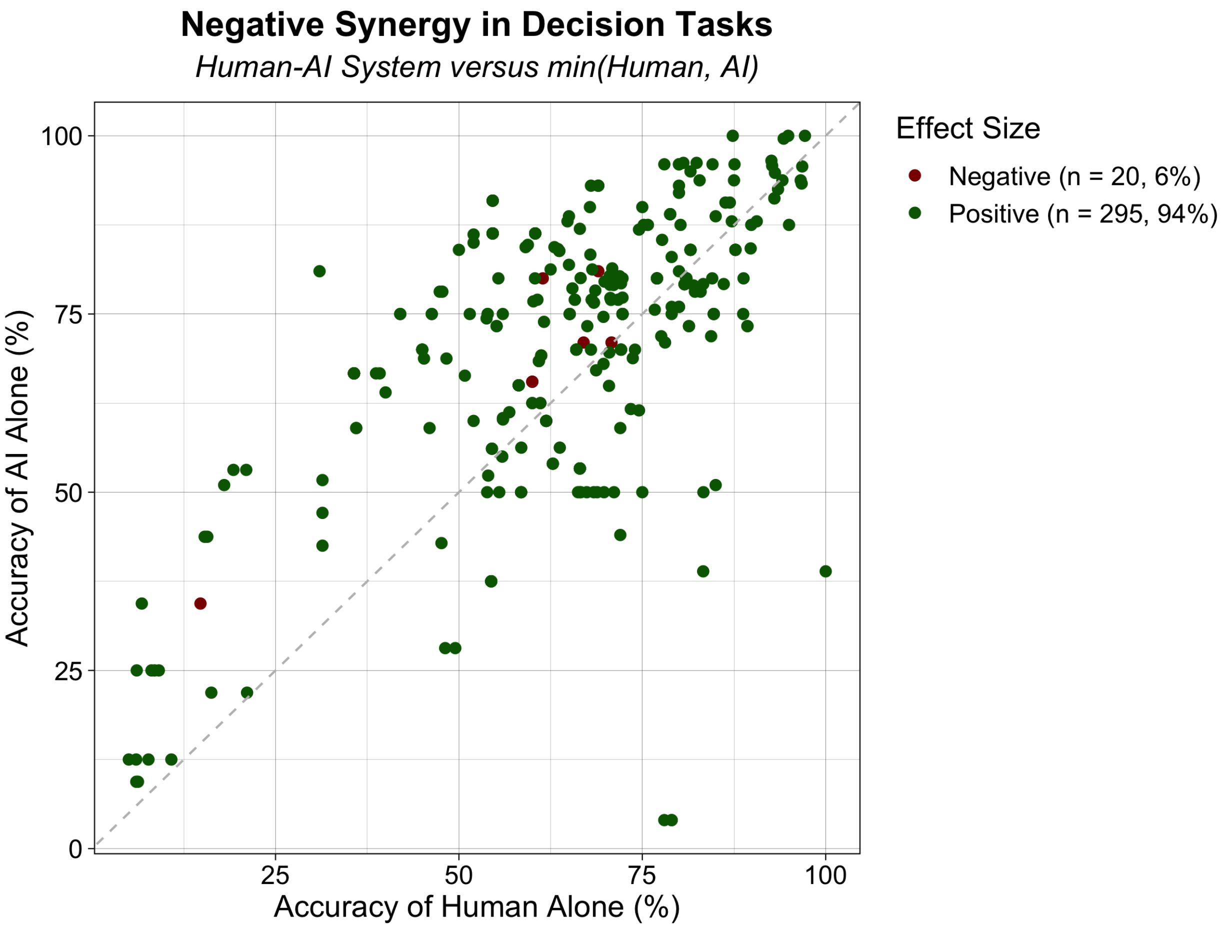

Figure S7: Scatterplot of the effect sizes that correspond to decision tasks where the researchers measured performance according to accuracy. The position of the points on the x-axis represents the accuracy of the human alone and the position of the points on the y-axis represents the accuracy of the AI alone. The colors of the points correspond to the value of the effect sizes, with negative effect sizes red and positive effect sizes green. A negative effect size means that the human-AI group performed worse than the AI alone and the human alone, so there was negative synergy, and a positive effect size means that the human-AI group performed better than the AI alone or the human alone, so there was not negative synergy. The gray dotted line indicates that the accuracy of the humans alone and AI alone are equivalent.

S2.6 Division of Labor

Only 3 of the 100+ experiments in our analysis explore processes with a pre-determined delegation of separate sub-tasks to humans and AI, so the difference between effect sizes with and without division of labor was not statistically significant. However, with the 4 effect sizes from these 3 experiments, we did find that positive human-AI synergy ($g = 0.22$, $t(104) = 0.69$, two-tailed $p = 0.494$, 95% CI -0.42 to 0.87) occurred among experiments with a predetermined division of labor between the human and AI, while in the experiments without this feature, the effect size was significantly negative ($g = -0.24$, $t(104) = -2.93$, two-tailed $p = 0.004$, 95% CI -0.40 to -0.08). For example, in Lee et al. (2021), physical therapists review patient-specific analysis and then provide feature-based feedback to an AI-system, which then generates an assessment for that patient based on its training data as well as this new information [6]. Here, the authors find that their human-AI system achieves

greater accuracy (93%) than either the therapist alone (75%) or AI alone without the therapist input (87%).

Hemmer at al. (2023) employ a different division of labor in an experiment involving image classification [7]. They develop an AI model that learns to both classify images and estimate human classifications of the same image. Then, for each image, this model classifies the images for which it has a higher confidence than it predicts the human would have and assigns the rest of the images to a human. These authors find that the human-AI system outperforms the human alone and AI alone (84% accuracy versus 67% and 75%, respectively).

Lastly, Lai et al. (2022) also create a pre-determined division of labor where an AI system generates a summary of a post, which a human can edit and refine [8]. They find, however, that the human alone produces higher quality summaries (5.8/7) than both the AI alone (4.7/7) and the human-AI combination (5.5/7). So this particular task allocation does not lead to human-AI synergy.

S2.7 Effect Sizes over Time

When we look at the evolution of human-AI synergy over the past years (see Figure S8), we observe potential signs of progress. This suggestive trend may reflect increased attention to designing studies that elicit human-AI synergy. Notably, however, the progress—if it exists—is not by any means rapid, and stands in stark contrast to the quick pace of development of AI systems themselves, particularly large language models (LLMs). We hope that our work can spur further progress in this domain.

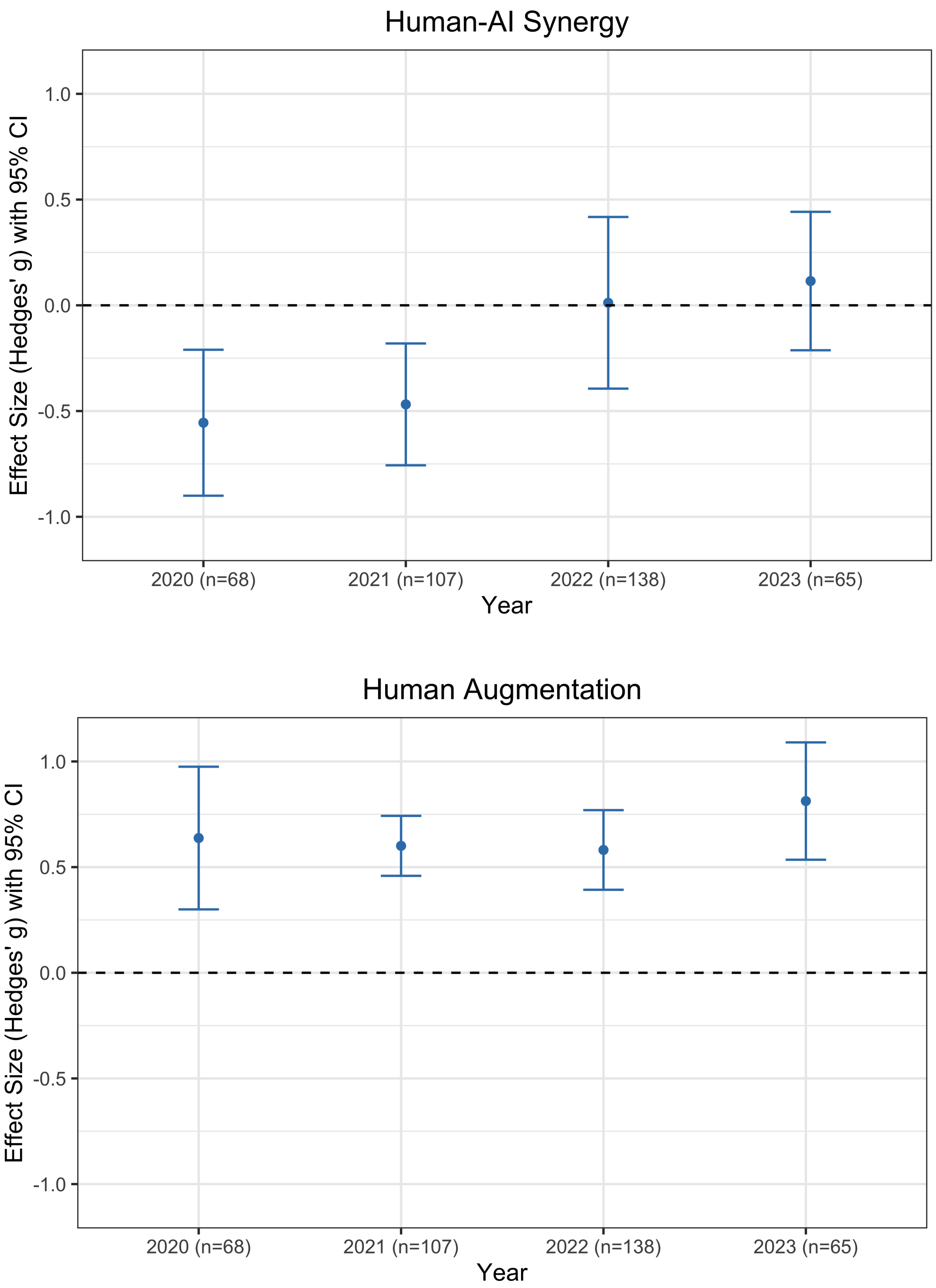

Figure S8: Plots of the meta-analytic average effect sizes for human-AI synergy and human augmentation by year of publication. The error bars correspond to 95% confidence intervals, and the dotted black line corresponds to an effect size of zero (no effect).

S2.8 Bias Tests

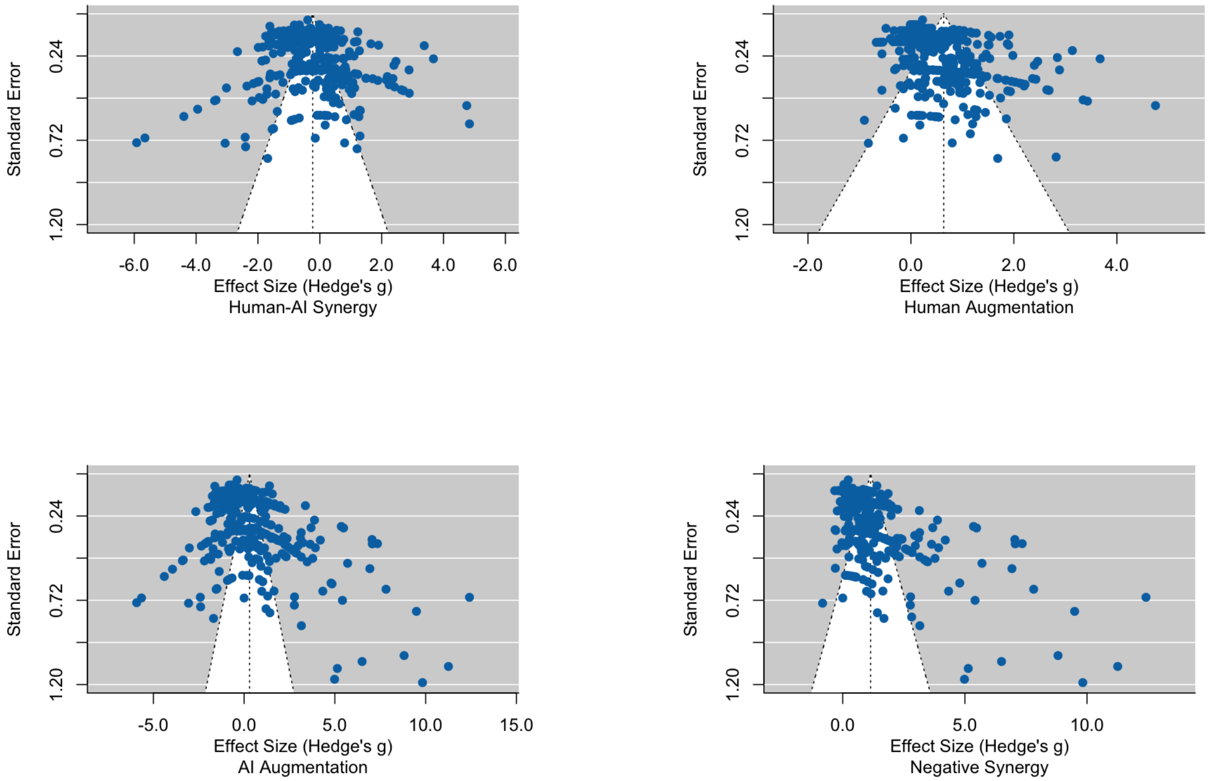

Figure S9: Funnel plots of the observed effect sizes and corresponding standard errors for human-AI synergy, human augmentation, AI augmentation, and negative synergy. The blue dots represent effect sizes and the vertical dotted black line represents the estimated pooled effect sizes from the meta-analytic models. The area shaded in white represents regions of statistical non-significance ($p > 0.05$), and the area shaded in gray represents regions of statistical significance ($p \leq 0.05$). In the absence of publication bias, we expect the points to fall roughly symmetrically around the estimated pooled effect size with a large proportion within the white triangle. We observe this pattern in the plot for human-AI synergy, but less so in the plots for human augmentation, AI augmentation, and negative synergy.

References

[1] Matthew J Page et al. “The PRISMA 2020 statement: an updated guideline for reporting systematic reviews”. In: *Bmj* 372 (2021).

[2] Julian PT Higgins, Tianjing Li, and Jonathan J Deeks. “Choosing effect measures and computing estimates of effect”. In: *Cochrane handbook for systematic reviews of interventions* (2019), pp. 143–176.

[3] Ankit Rohatgi. “Webplotdigitizer: Version 4.5”. In: *URL https://automeris. io/WebPlotDigitizer* 411 (2020).

[4] Jacob Cohen. *Statistical Power Analysis for the Behavioral Sciences.* Academic press, 2013.

[5] Vivian Lai et al. “Towards a Science of Human-AI Decision Making: An Overview of Design Space in Empirical Human-Subject Studies”. In: *Proceedings of the 2023 ACM Conference on Fairness, Accountability, and Transparency.* FAccT ’23. <conf-loc>, <city>Chicago</city>, <state>IL</state>, <country>USA</country>, </conf-loc>: Association for Computing Machinery, 2023, pp. 1369–1385. DOI: 10.1145/3593013.3594087. URL: https://doi.org/10.1145/3593013.3594087.

[6] Min Hun Lee et al. “A Human-AI Collaborative Approach for Clinical Decision Making on Rehabilitation Assessment”. In: *Proceedings of the 2021 CHI Conference on Human Factors in Computing Systems.* CHI ’21. <conf-loc>, <city>Yokohama</city>, <country>Japan</country>, </conf-loc>: Association for Computing Machinery, 2021. ISBN: 9781450380966. DOI: 10.1145/3411764.3445472. URL: https://doi.org/10.1145/3411764.3445472.

[7] Patrick Hemmer et al. “Human-AI Collaboration: The Effect of AI Delegation on Human Task Performance and Task Satisfaction”. In: *Proceedings of the 28th International Conference on Intelligent User Interfaces.* IUI ’23. Sydney, NSW, Australia: Association for Computing Machinery, 2023, pp. 453–463. DOI: 10.1145/3581641.3584052. URL: https://doi.org/10.1145/3581641.3584052.

[8] Vivian Lai et al. “An Exploration of Post-Editing Effectiveness in Text Summarization”. In: *Proceedings of the 2022 Conference of the North American Chapter of the Association for Computational Linguistics: Human Language Technologies.* Ed. by Marine Carpuat, Marie-Catherine de Marneffe, and Ivan Vladimir Meza Ruiz. Seattle, United States: Association for Computational Linguistics, July 2022, pp. 475–493. DOI: 10.18653/v1/2022.naacl-main.35. URL: https://aclanthology.org/2022.naacl-main.35.
