# Supplementary material for: When combinations of humans and AI are useful: A systematic review and meta-analysis
Source: Nat Hum Behav. 2024 Oct 28;8(12):2293–303. doi: 10.1038/s41562-024-02024-1 (PMC11659167; doi:10.1038/s41562-024-02024-1)
Supplement: Supplementary file 2 — Reporting Summary [file 41562_2024_2024_MOESM2_ESM.pdf]

nature portfolio

Corresponding author(s): Thomas Malone

Last updated by author(s): Aug 15, 2024

Reporting Summary

Nature Portfolio wishes to improve the reproducibility of the work that we publish. This form provides structure for consistency and transparency in reporting. For further information on Nature Portfolio policies, see our Editorial Policies and the Editorial Policy Checklist.

Statistics

For all statistical analyses, confirm that the following items are present in the figure legend, table legend, main text, or Methods section.

n/a	Confirmed	
☐	☒	The exact sample size (n) for each experimental group/condition, given as a discrete number and unit of measurement
☐	☒	A statement on whether measurements were taken from distinct samples or whether the same sample was measured repeatedly
☐	☒	The statistical test(s) used AND whether they are one- or two-sided<br>*Only common tests should be described solely by name; describe more complex techniques in the Methods section.*
☐	☒	A description of all covariates tested
☐	☒	A description of any assumptions or corrections, such as tests of normality and adjustment for multiple comparisons
☐	☒	A full description of the statistical parameters including central tendency (e.g. means) or other basic estimates (e.g. regression coefficient) AND variation (e.g. standard deviation) or associated estimates of uncertainty (e.g. confidence intervals)
☐	☒	For null hypothesis testing, the test statistic (e.g. F, t, r) with confidence intervals, effect sizes, degrees of freedom and P value noted<br>*Give P values as exact values whenever suitable.*
☒	☐	For Bayesian analysis, information on the choice of priors and Markov chain Monte Carlo settings
☒	☐	For hierarchical and complex designs, identification of the appropriate level for tests and full reporting of outcomes
☐	☒	Estimates of effect sizes (e.g. Cohen's d, Pearson's r), indicating how they were calculated

Our web collection on statistics for biologists contains articles on many of the points above.

Software and code

Policy information about availability of computer code

Data collection	We identified relevant studies through our systematic literature review, which we describe in our Methods section. We extracted relevant effect sizes and experiment characteristics from these studies, and we used Excel (Version 16.87) for the data collection.
Data analysis	We performed all quantitative analysis with the R statistical programming language in RStudio (Version 2023.06.0+421). We used the following packages: metafor (Version 4.6-0), tidyverse (Version 2.0.0), broom (Version 1.0.6), gtsummary (Version 2.0.0), forestploter (Version 1.1.2), and pacman (Version 0.5.1). We share the code used in analyze the data in our project's OSF repository (https://osf.io/wrq7c/?view_only=b9e1e86079c048b4bfb03bee6966e560).

For manuscripts utilizing custom algorithms or software that are central to the research but not yet described in published literature, software must be made available to editors and reviewers. We strongly encourage code deposition in a community repository (e.g. GitHub). See the Nature Portfolio guidelines for submitting code & software for further information.

Data

Policy information about availability of data

All manuscripts must include a data availability statement. This statement should provide the following information, where applicable:

- Accession codes, unique identifiers, or web links for publicly available datasets
- A description of any restrictions on data availability
- For clinical datasets or third party data, please ensure that the statement adheres to our policy

We compiled the data used in this analysis based on the studies identified in our systematic literature review. We make the data we collected available via the project's Open Science Framework repository (https://osf.io/wrq7c/?view_only=b9e1e86079c048b4bfb03bee6966e560). In our systematic review, we searched the following databases: the ACM Digital Library (ACM DL) (https://dl.acm.org/), Web of Science (https://clarivate.com/webofsciencegroup/solutions/web-of-science/), and Association for Information Systems eLibrary (AISeL) (https://aisnet.org/page/AISeLibrary).

Research involving human participants, their data, or biological material

Policy information about studies with human participants or human data. See also policy information about sex, gender (identity/presentation), and sexual orientation and race, ethnicity and racism.

Reporting on sex and gender	Our study consists of a systematic review and meta-analysis of prior work. We did not collect information on sex and gender from the original studies.
Reporting on race, ethnicity, or other socially relevant groupings	Our study consists of a systematic review and meta-analysis of prior work. We did not collect information on race, ethnicity, or other socially relevant groupings from the original studies.
Population characteristics	Our study consists of a systematic review and meta-analysis of prior work. We collected the following population characteristics from the original studies: number of participants, whether the participant was a crowdworker, and whether the participant had domain expertise for the task.
Recruitment	Our study consists of a systematic review and meta-analysis of prior work, so we did not recruit any participants.
Ethics oversight	Our study consists of a systematic review and meta-analysis of prior work, so it did not require ethical approval.

Note that full information on the approval of the study protocol must also be provided in the manuscript.

Field-specific reporting

Please select the one below that is the best fit for your research. If you are not sure, read the appropriate sections before making your selection.

☐ Life sciences ☒ Behavioural & social sciences ☐ Ecological, evolutionary & environmental sciences

For a reference copy of the document with all sections, see nature.com/documents/nr-reporting-summary-flat.pdf

Behavioural & social sciences study design

All studies must disclose on these points even when the disclosure is negative.

Study description	Our study consists of a systematic review and meta-analysis of prior experiments that evaluate human-AI systems. For each of these prior experiments, we collect quantitative data about the performance of the human alone, AI alone, and human-AI system on the task.
Research sample	Our research sample consists of experiments identified in our systematic literature review. Given the interdisciplinary nature of human-AI interaction studies, we performed this search in multiple databases covering conferences and journals in the computer sciences, information sciences, and social sciences, as well as other fields. Through consultation with a library specialist in these fields, we decided to target the Association for Computing Machinery Digital Library (ACM DL), Association for Information Systems eLibrary (AISeL), and the Web of Science Core Collection (WoS) for our review. To further ensure comprehensive coverage, we also conducted a forward and backwards search on all studies we found that meet our inclusion criteria. Our initial literature search yielded 5126 papers, and, per the review process described in the Methods Section, we identified 74 that met our inclusion criteria. These papers reported the results of 106 unique experiments, and many of the experiments had multiple conditions, so we collected a total of 370 unique effect sizes measuring the impact of human-AI collaboration on task performance. Our sample is thus representative of the past experiments involving human-AI systems. If there was bias in the individual studies or the publication process, our sample may not be representative of the general public. We conduct a number of bias tests to evaluate this possibility (see Methods Section) and discuss the results and implications in our paper.
Sampling strategy	As discussed above, given the interdisciplinary nature of human-AI interaction studies, we performed this search in multiple databases covering conferences and journals in the computer sciences, information sciences, and social sciences, as well as other fields. Through consultation with a library specialist in these fields, we decided to target the Association for Computing Machinery

Digital Library (ACM DL), Association for Information Systems eLibrary (AISeL), and the Web of Science Core Collection (WoS) for our review. To further ensure comprehensive coverage, we also conducted a forward and backwards search on all studies we found that meet our inclusion criteria.

Data collection

To calculate our primary outcome of interest -- the effect of combining human and artificial intelligence on task performance -- we recorded the averages and standard deviations of the task performance of the human alone, the AI alone, and the human and AI working with each other, as well as the number of subjects in each of these condition. We also considered and coded for multiple potential moderators of human-AI performance, namely: (1) publication date (2) pre-registration status (3) experimental design (4) data type (5) task type (6) task output (7) AI type (8) AI explanation (9) AI confidence (10) participant type and (11) performance metric. When collecting these data, the researchers were not blinded to the study hypotheses; however, we do not view this as a problem because the data extraction process was guided by a pre-registered protocol and relies entirely on published data.

Timing

To focus on current forms of artificial intelligence, we limited the search to studies published between January 1, 2020 and June 30, 2023. We conducted the search in July 2023.

Data exclusions

Per our pre-registration (see https://osf.io/wrq7c/?view_only=b9e1e86079c048b4bfb03bee6966e560), we applied the following criteria to select studies that fit our research questions. First, the paper needed to present an original experiment that evaluates some instance in which a human and an AI system work together to perform a task. Second, it needed to report the performance of (1) the human alone, (2) the AI alone, and (3) the human-AI system according to some quantitative measure(s). As such, we excluded studies that reported the performance of the human alone but not the AI alone, and likewise we excluded studies that reported the performance of the AI alone but not the human alone. Following this stipulation, we also excluded purely meta-analyses and literature reviews, theoretical work, qualitative analyses, commentaries, opinions, and simulations. Third, we required the paper to include the experimental design, the number of participants in each condition, and the standard deviation of the outcome in each condition, or enough information to calculate it from other quantities. Finally, we required the paper to be written in English. See our PRISMA Flow Diagram (Figure S1) for the number of studies excluded in the systematic review and meta-analysis and rationale for exclusion.

Non-participation

We did not recruit participants in our study.

Randomization

Our study consists of a systematic review and meta-analysis of prior work, so randomization was not applicable in our study. The data in our meta-analysis cover all of experiments identified in our systematic review.

Reporting for specific materials, systems and methods

We require information from authors about some types of materials, experimental systems and methods used in many studies. Here, indicate whether each material, system or method listed is relevant to your study. If you are not sure if a list item applies to your research, read the appropriate section before selecting a response.

Materials & experimental systems

n/a	Involved in the study	
☒	☐	Antibodies
☒	☐	Eukaryotic cell lines
☒	☐	Palaeontology and archaeology
☒	☐	Animals and other organisms
☒	☐	Clinical data
☒	☐	Dual use research of concern
☒	☐	Plants

Methods

n/a	Involved in the study	
☒	☐	ChIP-seq
☒	☐	Flow cytometry
☒	☐	MRI-based neuroimaging
